# Baseline Characteristics of Individuals with Metastatic Cancer Enrolled in the Alberta Cancer Exercise Study and 12-Week Findings for Symptom-Related and Physical Fitness Measures

**DOI:** 10.3390/curroncol32100560

**Published:** 2025-10-07

**Authors:** Shirin M. Shallwani, S. Nicole Culos-Reed, Kerry S. Courneya, Tanya Williamson, Christopher Sellar, Harold Lau, Anil Abraham Joy, Jacob C. Easaw, Michelle Audoin, Edith Pituskin, Margaret L. McNeely

**Affiliations:** 1 Faculty of Rehabilitation Medicine, University of Alberta, 2-50 Corbett Hall, Edmonton, AB T6G 2G4, Canada; sshallwa@ualberta.ca; 2 Faculty of Kinesiology, University of Calgary, 2500 University Dr NW, Calgary, AB T2N 1N4, Canada; nculosre@ucalgary.ca (S.N.C.-R.); willt@ucalgary.ca (T.W.); 3 Faculty of Kinesiology, Sport, and Recreation, University of Alberta, 1-113 University Hall, Edmonton, AB T6G 2H9, Canada; kerry.courneya@ualberta.ca; 4 Department of Physical Therapy, University of Alberta, 2-50 Corbett Hall, Edmonton, AB T6G 2G4, Canada; csellar@ualberta.ca; 5 Department of Oncology, University of Calgary, 3395 Hospital Drive NW, Calgary, AB T2N 5G2, Canada; hlau@ucalgary.ca; 6 Medical Oncology, Cross Cancer Institute, Alberta Health Services, 11560 University Avenue, Edmonton, AB T6G 1Z2, Canada; anil.joy@ahs.ca (A.A.J.); jay.easaw@ahs.ca (J.C.E.); 7 Department of Oncology, University of Alberta, 11560 University Avenue NW, Edmonton, AB T6G 1Z2, Canada; 8 Patient Partner, Cancer Rehabilitation Clinic, University of Alberta; 2-50 Corbett Hall, Edmonton, AB T6G 2G4, Canada; 9 Faculty of Nursing, University of Alberta, 3-141 Edmonton Clinic Health Academy, Edmonton, AB T6G 1C9, Canada; pituskin@ualberta.ca; 10 Cross Cancer Institute, 11560 University Avenue, Edmonton, AB T6G 1Z2, Canada

**Keywords:** metastatic cancer, advanced cancer, exercise, implementation, community-based, adverse event

## Abstract

Exercise has been found to be safe and beneficial for people with advanced cancers, but more research is needed to understand how best to design and implement exercise programming. The Alberta Cancer Exercise (ACE) study examines the effectiveness and implementation of a 12-week community-based exercise program in Alberta, Canada, for people diagnosed with cancer. Here, we describe the characteristics of individuals with metastatic cancer enrolled in the ACE program and report 12-week changes in self-reported and objective outcomes. Of 306 participants, 274 (89.5%) completed the 12-week study. Many participants were female (65.4%), with ≥1 comorbidity (71.9%), and on active cancer treatment (74.8%). Common cancer types included breast (33.7%), genitourinary (16.7%), and digestive (15.0%). Frequent sites of metastasis were bone (44.8%), liver (28.8%), and lung (25.8%). The mean exercise attendance rate was 73.6%. One exercise-related adverse event (0.3%) and one non-exercise-related adverse event (0.3%) occurred, both in individuals with brain metastases. Participants demonstrated strong interest and engagement in exercise, with significant improvements in weekly physical activity, symptoms, quality of life, and physical fitness. Greater benefits were found among subgroups of male participants, those not undergoing chemotherapy, and those receiving group personal training or virtual circuit training. A low rate of adverse events is anticipated.

## Simple Summary

Exercise can help people with advanced cancers feel better and improve their quality of life, but there is limited information on how best to deliver safe and effective programs. This study looked at a 12-week community exercise program in Alberta, Canada, designed for people with cancer, including those with metastatic disease. Most participants with metastatic cancer completed the program and attended regularly. Exercise was well tolerated, with a very low incidence of adverse events. Safety was supported through screening/triage, check-ins, and supervised exercise. Participants experienced meaningful improvements in physical activity levels, symptoms, overall wellbeing, and physical fitness. Some groups, such as people not receiving chemotherapy, male participants, and those in group personal training or online classes, experienced even greater benefits. These findings show that community-based exercise programs are safe and helpful for people with metastatic cancer. This work provides important direction for expanding access to exercise programs and shaping future cancer care.

## 1. Introduction

Advanced cancers, including metastatic cancers, typically involve cancer spread and require long-term therapies for cancer control and symptom palliation [1]. Although survival rates are improving due to progress in treatment options, people diagnosed with advanced cancers frequently face diverse cancer-specific and treatment-related challenges. These include, but are not limited to, uncertain prognosis, high symptom burden, decreased physical function, and psychosocial concerns [2,3,4,5]. As such, there is a tremendous impact of advanced cancer upon daily functioning and quality of life [4,6,7,8].

Exercise has been found to be safe, feasible, and associated with important health benefits for people with advanced cancer [9,10,11,12,13,14]. Structured exercise interventions can help improve quality of life, alleviate cancer-related symptoms (e.g., fatigue, dyspnea, insomnia), and enhance physical function in these individuals [9,10,13,15]. However, people with advanced cancer often experience barriers to exercise participation. Reported challenges include symptoms of fatigue, pain, and shortness of breath, apprehensions about injury, limited professional guidance, and lack of accessible, suitable settings [2,16,17,18,19,20,21]. Current exercise oncology research remains limited in applicability to the advanced cancer population [10,22,23,24]. There is a need for larger, well-designed studies examining evidence-based exercise interventions that can be tailored to different cancer populations, that focus on reducing burden (e.g., travel) and optimizing adherence, and that can be successfully implemented in accessible, real-world settings [9,10,13,15,21].

The Alberta Cancer Exercise (ACE) study is a hybrid effectiveness–implementation study, designed to investigate the effectiveness of a 12-week exercise program for cancer survivors and to examine real-world implementation outcomes [25,26]. To address gaps in cancer care services, further work is needed to investigate how best to adapt and implement evidence-based exercise programs specifically for people with advanced cancer within cancer institutions and community settings across Canada. While exercise has generally been found to be safe and feasible in the advanced cancer population, it has not been well explored within the context of community-based settings. In studies of community- and home-based walking programs, there have been challenges identified with recruiting and retaining participants [27,28]. Given the heterogeneity of the advanced cancer population and the diverse challenges experienced by individuals, there is a need for large-scale research specifically related to community-based exercise, with consideration given to outcomes that are important to this population, particularly cancer-related symptoms and functional ability [29]. Findings from the ACE study can help elucidate the characteristics of people with metastatic cancer who choose to participate and are able to complete the program, and the relative benefit from exercise participation. Thus, the ACE study provides the opportunity to further examine the feasibility and effectiveness of evidence-based community-based exercise programs among individuals with metastatic disease.

**Objectives:** The primary objective of this study is to describe the baseline characteristics of individuals with metastatic cancer who enrolled in the 12-week community-based ACE program. Secondary objectives are to examine the feasibility of the program (i.e., safety, completion, and attendance) and to evaluate 12-week changes in physical activity behavior, symptoms, quality of life, and physical fitness measures.

## 2. Materials and Methods

### 2.1. Study Design and Participants

The ACE study is a hybrid effectiveness–implementation trial evaluating a 12-week cancer-specific, community-based exercise program delivered in Alberta, Canada. The ACE trial commenced in January 2017 and closed to recruitment in February 2023. The methods and initial findings of the ACE study have been previously reported in detail [25,26]. Participants enrolled in the ACE study include adults with any type and stage of cancer, at different points along the cancer trajectory (up to three years following completion of cancer treatment).

The current study consists of a secondary analysis of baseline and 12-week data for a subgroup of participants with metastatic cancer enrolled in the ACE study. Participants were identified if they self-reported disease spread on their pre-exercise screening form. Metastatic disease was confirmed through the medical clearance process or on medical chart review. For the present analyses, participants with primary bone cancer or multiple myeloma were excluded.

### 2.2. Screening Process

Due to the implementation focus of the ACE study, the study procedures were adapted and refined overtime to improve participant screening, enhance study feasibility, and reduce participant burden [25]. The screening process also helped inform the type of group-based exercise training that was recommended for each participant (i.e., in-person circuit training, virtual circuit training, or personal training). There were four key steps in the screening and triage of ACE participants:

(1) Pre-screening to identify participants with high-risk cancers (e.g., lung, neurological, pancreatic) and/or metastatic disease spread.
(2) Cancer intake form and Physical Activity Readiness Questionnaire for Everyone (PAR-Q+) to identify specific cancer-related concerns and other health conditions potentially impacting physical activity participation.
(3) In-person or telephone interview with a clinical exercise physiologist to review screening findings and to determine need for medical clearance and/or specialized support.
(4) Physical fitness assessment to evaluate physical function and mobility and to identify participants with underlying issues requiring medical clearance and/or specialized support.

### 2.3. Program Characteristics

The ACE program involves full-body, group-based circuit training or personal training, with a combination of aerobic, resistance, balance, and flexibility exercises. Exercise sessions consist of ≥60 min of mild- to moderate-intensity exercise, offered twice a week for 12 weeks. Prior to COVID-19, sessions were delivered and supervised by ACE-trained exercise specialists at 18 urban sites across the province of Alberta, Canada, including three academic institutions, six YMCA locations, three Wellspring Alberta locations, and six municipal fitness centers. With the onset of the COVID-19 pandemic in March 2020, exercise programming was delivered in a virtual format. In-person assessments and exercise sessions gradually resumed starting in September 2021 with 13 in-person sites restored, and virtual sessions continued to be offered for the remainder of the ACE study. Program details have been outlined in detail in a previous paper [26].

### 2.4. Data Collection

The main study outcomes include physical activity level, physical fitness, cancer-related symptoms, and health-related quality of life. Outcomes are evaluated at baseline, at 12 weeks (immediately post-program), and at one year. Data from the ACE study are stored securely in a REDCap database housed in the Faculty of Medicine and Dentistry at the University of Alberta.

### 2.5. Participant Characteristics

To better understand the profile of the ACE participants with metastatic cancer, we extracted data on the following baseline characteristics that were collected across all participants over the study duration:

(1) Sociodemographic factors: Age, biological sex, gender identity, ethnicity, marital status, education level, income, employment status, smoking status, drinking status
(2) Anthropometric measures: Body mass index
(3) Medical and cancer history: Number of comorbidities, primary cancer type, number and location of metastases, treatment status, current and completed treatments
(4) Exercise-related outcomes: Physical Activity Stages of Change, Godin-Shephard Leisure-Time Physical Activity Questionnaire (to calculate physical activity minutes per week [30])
(5) Symptom-related and quality of life measures: Functional Assessment of Cancer Therapy-Fatigue (FACT-F; also referred to as FACIT-F) scale (higher FACT scores indicate better quality of life), Edmonton Symptom Assessment Scale (ESAS; higher ESAS scores indicate higher symptom burden), 5-level EQ-5D (EQ-5D-5L; higher levels on EQ-5D-5L dimensions indicate more severe problems), EQ visual analogue scale (EQ VAS; higher EQ VAS scores indicate better health)
(6) Physical fitness measures: 30 s timed sit-to-stand (in-person and virtual assessments), one-legged stance (in-person and virtual assessments), 6-Minute Walk Test (6MWT) (in-person assessments only; discontinued during the COVID-19 pandemic), 2 min step test (included in virtual assessments to replace the 6MWT during the COVID-19 pandemic), active shoulder flexion range of motion (in-person and virtual assessments)

We also extracted 12-week data for physical activity minutes per week, symptom-related and quality of life measures, as well as physical fitness measures.

### 2.6. Data Analysis

Descriptive statistics were used to report sociodemographic, cancer-related, and exercise-related characteristics, symptom-related measures, and physical fitness measures of the ACE participants with metastatic cancer at baseline. Prior to analysis, we explored missing data and outliers and examined test assumptions of normality. To check for normality in the distribution of the study data, we used the Kolmogorov–Smirnov statistical test for large sample sizes and graphed the data for each study outcome [31].

The primary self-reported outcome measure was the FACT-F questionnaire, which was examined as total FACT-F scale and FACT Trial Outcome Index (TOI) scores. In particular, the TOI measure has been used to assess health-related quality of life in people with cancer and incorporates measures of physical wellbeing, functional wellbeing, and cancer-specific symptoms (i.e., fatigue) [32]. The TOI has been found to be associated with progression-free survival in a large cohort of individuals with advanced cancer [33]. For this analysis, we also examined the FACT-G scale and FACT-Fatigue subscale scores. To explore symptom burden in the participants, we descriptively analyzed ESAS scores as: (a) individual symptom scores (0–10); (b) total symptom distress scores (0–90); (c) physical scores, (0–60); and (d) emotional scores (0–20) [34]. We also categorized the severity of individual symptoms using the following ESAS score categories: (a) no/mild symptoms (score 0–3); (b) moderate symptoms (score 4–6); and (c) severe symptoms (score 7–10) [34,35]. Similarly, we descriptively analyzed the EQ-5D-5L data for each dimension according to problem severity category: (a) no problems (Level 1); (b) slight problems (Level 2); (c) moderate problems (Level 3); (d) severe problems (Level 4); and (e) extreme problems (Level 5) [36]. To further explore physical fitness levels, baseline participant data for the 30 s sit-to-stand, one-legged stance, and strength measures were compared to age- and sex-matched norms [37,38,39,40].

For study feasibility, we determined the overall study enrollment and 12-week completion rates for the participants with metastatic cancer in the ACE study. Comparisons of baseline characteristics between participants who remained in the study vs. participants who dropped out (including those who withdrew from the study and those who were lost to follow-up prior to the 12-week study time point) were conducted using independent samples t-tests and Mann–Whitney U tests for continuous data, and chi-squared tests for categorical data. Implementation feasibility of the ACE program was examined through calculating rates of attendance in the exercise program (number of exercise sessions attended/number of sessions offered in program) for all participants included in the analysis [41]. Based on the previous literature examining supervised exercise programs with cancer and older adult populations [10,11,42,43,44], the target attendance rate for this study was defined as participation in at least 70% of the sessions over the 12-week exercise program. The feasibility of the community-based ACE program was considered acceptable if at least 60% of the participants with metastatic cancer met the study target rate of 70% for exercise program attendance. Safety of the ACE program was examined by reporting the number and severity of exercise-related adverse events that occurred in the participants during the 12-week program. The severity of any adverse event was rated according to Common Terminology Criteria for Adverse Events system [45]. A very low rate (<1%) of adverse events was considered acceptable for the ACE program. The rates for safety and feasibility were established a priori in the study protocol [25].

**Effects of exercise participation:** Paired Student’s t-tests and Wilcoxon signed-rank tests for non-parametric data were used to analyze short-term (12-week) changes in physical activity, symptoms, quality of life, and physical fitness measures following participation in the ACE program. We also explored participant outcomes by subgroups of biological sex, current chemotherapy status, and exercise training type (in-person personal vs. in-person circuit vs. virtual circuit training).

Clinically meaningful improvements for selected patient-reported outcomes and physical fitness measures were analyzed based on the following minimal important differences (MIDs): 5–6 points on the FACT TOI, 3 points on the FACT Fatigue subscale, and 3–5 points on the FACT-G scale, corresponding to an estimated 6- to 8-point MID for the combined total FACT-F scale score (FACT-G scale plus Fatigue subscale items) [32,46,47]; 3 points, 2 points, and 3 points on the ESAS physical, emotional, and total symptom distress scores, respectively, and 1 point on the ESAS wellbeing item score [48,49]; and 5 points on the EQ VAS score [50]. A 10% increase was used for physical activity minutes per week [25]. For physical fitness measures, established MIDs were 2 repetitions for the 30 s sit-to-stand test [51,52]; 24 s for one-legged stance [53]; 30 m for the 6 min walk test [54]; 10% increase for the 2 min step test [55,56]; and 14 degrees for shoulder range of motion [57].

## 3. Results

### 3.1. Participant Flow

**Figure 1:**
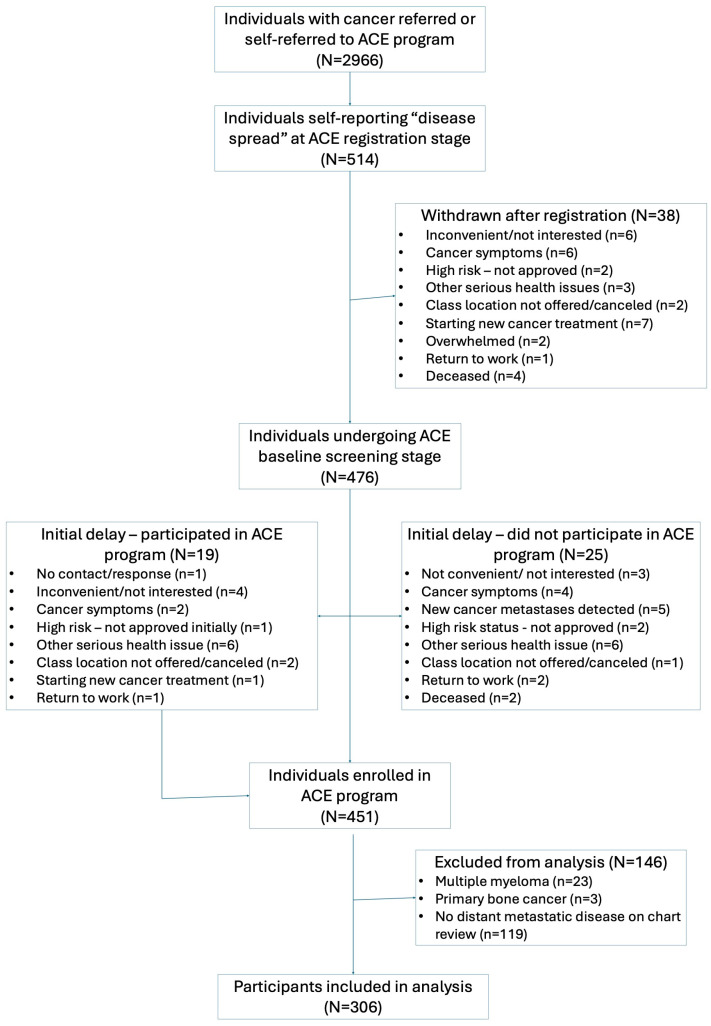
Flow chart for Alberta Cancer Exercise participants with metastatic cancer included in secondary analysis.

Of 2966 participants referred to the ACE program between 2017 and 2023, 306 participants (10.3%) enrolled in the study had confirmed presence of metastatic cancer and were included in this secondary analysis (Figure 1). Of 306 participants with metastatic cancer, 274 (89.5%) completed the 12-week ACE study. Reasons for dropout in 32 participants included cancer progression/recurrence (n = 14), difficulty with attending program due to symptoms, commute, or family responsibilities (n = 3), other health illness (n = 3), or no response/reason given (n = 12). Due to the COVID-19 initial lockdown in March 2020 and lack of staff access to research and clinical sites, in-person post-intervention study assessments were not conducted for all participants completing the Winter 2020 session (n = 30). This contributed to missing data in physical fitness measures, before virtual study assessments were implemented for participants joining after May 2020.

The study completion rates by region were 92.1% for Calgary, 87.7% for Edmonton, 85.7% for the North Zone, and 86.7% for the South Zone. For female and male participants, the rates were 90.5% and 87.7%, respectively. By age group, the study completion rates were 87.0% for young adults, 88.5% for middle-aged adults, and 92.4% for older adults. The completion rates by exercise training type were 87.2% for in-person circuit training, 89.0% for in-person personal training, and 94.5% for virtual circuit training.

### 3.2. Baseline Characteristics

Baseline sociodemographic and cancer-related characteristics of the participants are provided in Table 1 and Table 2. Briefly, many ACE participants were female (65.4%), middle-aged (62.4%), married or common-law (73.9%), of European descent (75.8%), and diagnosed with at least one comorbidity (71.9%). The most common primary cancer types were breast (33.7%), genitourinary (16.7%), digestive (15.0%), and lung (10.8%). Frequent sites of metastases included bone (44.8%), liver (28.8%), lung (25.8%), and other (20.3%). Most participants (74.8%) were on active cancer treatment at the time of their enrolment in the ACE program, with chemotherapy (35.9%) and hormone therapy (28.4%) being the most common current therapies. Reasons for the 77 participants being off treatment included: surveillance (45.5%), remission (50.6%), no further treatment (1.3%), and treatment stopped due to side effects (2.3%).

Exercise-related characteristics of the ACE participants with metastatic cancer are provided in Table 3. At baseline, the participants were engaging in a mean of 90.2 min of physical activity per week. About half of the participants (49.3%) were classified as sedentary in terms of their physical activity level, and over half were in the contemplation (23.2%) or preparation (31.4%) stages of physical activity behavior change.

Baseline symptom and physical fitness measures are provided in Supplementary File S1. Approximately half of the participants (49.0%) were below age- and sex-matched norms for the one-legged stance measure, while 79.4% were below norms for the 30 s sit-to-stand measure. Mean scores for individual ESAS items (Supplementary File S2) ranged from 0.6 to 3.5, with better scores for nausea, appetite, and shortness of breath (mean scores: 0.6, 1.1, and 1.2, respectively), and worse scores for tiredness and wellbeing (mean scores: 3.5 for both). Most participants (99.0%) reported no or slight problems with self-care on the EQ-5D-5L (Supplementary File S3). Activity was identified as most troublesome on the EQ-5D-5L with almost 30% of participants indicating moderate, severe, or extreme problems.

**Comparisons by study completion:** Baseline characteristics by study completion status are also presented in Table 1, Table 2 and Table 3 and Supplementary File S1. Statistical comparisons of baseline characteristics demonstrated that participants who dropped out of the ACE study were more likely to be receiving current chemotherapy (*p* = 0.011) and had higher rates of lung metastasis (*p* = 0.043). Moreover, study dropouts had lower rates of college or university level education (*p* = 0.035) and of being in the action/maintenance stages of physical activity change (*p* = 0.032) than those who completed the 12-week study. Study completers were more likely to be receiving hormone therapy (*p* = 0.035) and have other types of comorbidities (*p* = 0.032), and also had better baseline scores on the FACT-G scale (*p* = 0.001), FACT TOI (*p* = 0.009), FACT Fatigue subscale (*p* = 0.027), FACT-F scale (*p* = 0.003), ESAS physical (*p* = 0.004), ESAS total (*p* = 0.009), and EQ VAS (*p* < 0.001) compared to dropouts. There were no differences in physical activity minutes per week (*p* = 0.580) or physical fitness measures (*p* > 0.05) at baseline.

### 3.3. Program Feasibility and Safety

**Exercise location and type:** The ACE participants included in this analysis enrolled in exercise classes at the following study locations: Edmonton (n = 130, 42.5%), Calgary (n = 140, 45.8%), North Zone (Fort McMurray: n = 2, 0.7%; Grand Prairie: n = 8, 2.6%; Red Deer, n = 11, 3.6%), and South Zone (Lethbridge: n = 6, 2.0%; Medicine Hat: n = 9, 2.9%). The types of group-based exercise classes attended by the participants included in-person circuit training (n = 133, 43.5%), in-person personal training (n = 100, 32.7%), and virtual circuit training (n = 73, 23.9%).

**Exercise session attendance:** The mean percentage of exercise sessions attended by the participants over the 12-week ACE program was 73.7% (based on n = 301; missing data for n = 5 participants in the North Zone). By location, the 12-week program attendance rates were 71.0% for Calgary, 76.5% for Edmonton, 71.6% for the North Zone (Fort McMurray: N/A; missing data for 2 participants; Grand Prairie: 78.8%; missing data for 3 participants; Red Deer: 68.3%), and 75.8% for the South Zone (Lethbridge: 81.8%; Medicine Hat: 71.7%). Mean program attendance rates by biological sex and by age group were 72.3% in female participants, 76.3% in male participants, 67.1% in young adults, 72.3% in middle-aged adults, and 78.0% in older adults. By exercise training type, attendance rates were 69.8% for in-person circuit training, 77.1% for in-person personal training, and 75.7% for virtual circuit training. Of the 301 participants with data on exercise attendance, 65.1% met the target rate of 70% attendance.

**Adverse events:** There were two adverse events which occurred during participation in the ACE program, resulting in a rate of adverse events of 0.65%. The incidents consist of one exercise-related event (CTCAE Grade 1: nervous system disorder) and one minor non-exercise-related event (CTCAE Grade 1: injury). The first event involved a participant with brain metastases who experienced an absence seizure while resting (seated) between exercise sets at a community site. The event lasted a short duration (<10 s) and resolved on site. The participant was immediately taken to the hospital for further investigation. It was later determined that the participant had progression of brain metastases, and they were withdrawn from ACE by their oncology care team. The second case was a participant with brain metastases who experienced a fall resulting in a hit to the head. The fall occurred prior to start of the exercise session in another location of the community-based site. The participant was taken to the hospital for further investigation and was cleared with no resulting injuries.

### 3.4. Changes in Symptom-Related, Quality of Life, and Physical Fitness Measures

Baseline and 12-week scores for the symptom-related, quality of life, and physical fitness measures are presented in Table 4. On the FACT measure, there were statistically significant improvements on the TOI, Fatigue subscale, and FACT-F scale scores in the ACE participants who completed the 12-week study (*p* < 0.001), and a significant but weaker effect on the FACT-G scale score (*p* = 0.010). No significant changes were found on the ESAS scores in the participants, but statistically and clinically significant improvements were found for the EQ VAS score at 12 weeks (*p* < 0.001). Physical activity minutes per week increased significantly (*p* < 0.001) and clinically from baseline to 12 weeks. In terms of physical fitness measures, there were significant improvements for all measures from baseline to 12 weeks (*p* < 0.001), with clinically significant improvements on the 30 s sit-to-stand test, 6MWT, and 2 min step test. The proportions of responses by level of severity on the EQ-5D-5L dimensions for the ACE participants who completed the 12-week study are presented in Supplementary File S4.

**Subgroup analyses:** Pre–post program changes in the symptom-related and physical fitness measures for subgroups by biological sex and chemotherapy status are presented in Supplementary Files S5 and S6. Male participants with metastatic cancer demonstrated statistically and clinically significant improvements over 12 weeks on the FACT TOI and Fatigue subscale (all *p* < 0.001) and statistically significant improvement on the FACT-G (*p* = 0.018) and FACT-F (*p* < 0.001) scores. Female participants showed significant benefits for the FACT Fatigue subscale (*p* < 0.001), TOI (*p* = 0.002) and FACT-F (*p* = 0.006) scores, but these changes did not meet clinically significant levels. ESAS scores did not change in either group over 12 weeks, but there was statistically and clinically significant improvement of the EQ VAS score in both groups (*p* < 0.001). Statistically and clinically meaningful benefits were found in both groups for physical activity minutes per week, 30 s sit-to-stand, 6MWT, and 2 min step test (*p* < 0.001), while statistically significant benefits were seen for the one-legged stance (*p* < 0.001) and bilateral shoulder range of motion (*p* < 0.001 to *p* = 0.036) tests.

With regard to chemotherapy status, participants who were off chemotherapy treatment demonstrated significant improvements at 12 weeks on the FACT and EQ VAS scores (*p* < 0.001), with both statistically and clinically meaningful improvements on the FACT Fatigue subscale and EQ VAS scores. For those receiving chemotherapy, there were no statistical or clinical improvements on symptom-related and quality of life measures. There were no changes on ESAS scores in those who were off chemotherapy. ESAS physical and total symptom distress scores statistically worsened in participants on chemotherapy (*p* = 0.036 and *p* = 0.031), but these changes did not reach clinically significant levels. Participants both on and off chemotherapy showed significant and clinically meaningful increases in weekly minutes of physical activity (*p* < 0.001). Measures of physical fitness improved significantly in the participants who were not receiving chemotherapy (*p* < 0.001), with clinical improvements in 30 s sit-to-stand, 6MWT, and 2 min step test. Participants receiving current chemotherapy improved significantly on the 30 s sit-to-stand, one-legged stance, and 6MWT at 12 weeks (*p* < 0.001), with clinical improvements seen in the 30 s sit-to-stand and 6MWT.

Changes in the symptom-related and physical fitness measures by exercise training type are presented in Supplementary File S7. ACE participants taking part in in-person personal and virtual circuit training improved statistically on all FACT measures (*p* < 0.001 to *p* = 0.046), while those in in-person circuit training improved statistically on the Fatigue subscale measure only (*p* = 0.022). Clinically meaningful improvement was seen on the FACT TOI score for in-person personal training participants. There were no significant changes on the ESAS scores for any of the three groups, except that in-person circuit training participants had worse ESAS physical scores (*p* = 0.039) and in-person personal training participants had better ESAS physical scores (*p* = 0.031) at 12 weeks. EQ VAS, physical activity minutes per week, 30 s sit-to-stand test, and left shoulder range statistically improved in all three groups. Participants in the two in-person groups significantly improved on the one-legged stance and 6MWT measures (*p* < 0.001; *note: 6MWT was measured only in virtual participants able to attend in-person testing). Virtual group participants showed improvements for the 2 min step test (*p* < 0.001; *note: 2 min step test was not part of the test battery for in-person participants). Left shoulder range of motion improved significantly in all three groups (*p* = 0.007 to *p* = 0.034), while right shoulder improved in virtual circuit participants only (*p* = 0.002).

## 4. Discussion

This study provides insight on the effectiveness and implementation of community-based exercise programming for individuals with metastatic cancer. The findings demonstrate that many people diagnosed with advanced cancers were willing and able to access, successfully participate in, and benefit from the exercise program. To our knowledge, this analysis from the ACE study represents one of the largest study cohorts of people with metastatic cancer in the exercise oncology literature to date [9,13,15,24]. Individuals with confirmed metastatic cancer at study entry made up close to 12% of the total ACE study cohort [26]. The high completion and attendance rates of ACE participants with metastatic cancer support the overall feasibility of a community-based exercise program model of care. Moreover, exercise participation resulted in significant improvements in physical activity, symptoms, quality of life, and physical fitness measures, supporting the benefit of exercise. Individuals with metastatic cancer commonly face barriers to exercise, such as cancer-related symptoms and concerns, as well as lack of accessible information and support [18,19,20,21]. Key features of the ACE program, including the community-based in-person and virtual offerings, option for self-referral, and tailored exercise support, likely contributed to addressing some of these barriers and supporting access to exercise programming for these individuals. Given the rising prevalence of people living with metastatic cancer and the complex challenges they experience, tailored and accessible exercise programs, as well as other supportive care services, are needed to meet the unique needs of this population [1,58,59].

Consistent with the full ACE cohort at baseline [26], breast cancer was the most common diagnosis in 34% of the subgroup of participants with metastatic cancer. However, this percentage of breast cancer was lower than the full ACE cohort (45%). Moreover, our sample included over 200 participants (66.3%) with a non-breast cancer diagnosis. A recent estimation of the number of individuals living with metastatic cancer in the U.S. indicated high prevalence rates for lung, breast, colorectal, and prostate cancers [58]. In a Canadian study of 1366 individuals with metastatic cancer referred to an out-patient palliative care clinic, gastrointestinal cancer was the most common cancer type [60]. These diagnoses are consistent with the characteristics of the ACE metastatic subgroup which showed breast, genitourinary (e.g., prostate), digestive (e.g., gastrointestinal), and lung cancer as the most common cancer types. Due to the heterogeneity of the metastatic cancer population in terms of cancer types and treatment regimens, people diagnosed at this stage of disease experience varying prognostic estimates, physical impairments, and symptom-related issues [1,4]. However, there is increasing evidence of exercise benefits for a variety of advanced and metastatic cancers including breast, lung, prostate, and colorectal cancers [15]. These findings further highlight the need for community-based programming that can support exercise participation in people with both these common as well as less common types of metastatic cancer.

While the ACE study was feasible for a large, diverse group of individuals with metastatic cancer, our analysis revealed that other cancer-related factors were related to study completion status and changes in study outcomes. Participants who dropped out of the study were more likely to have lung metastases and be undergoing chemotherapy at the time of enrollment. These findings suggest individuals with specific disease characteristics may require further tailored support to sustain participation in exercise programs. A key finding of our study is that ACE participants receiving chemotherapy who remained in the study improved their physical activity levels and physical fitness measures without a negative impact on many of the self-reported measures of quality of life and fatigue. Although they did not experience the improvements seen in participants who were off chemotherapy, particularly for the ESAS physical and total symptom scores, the results show that overall, their wellbeing and symptoms did not worsen to a clinically significant extent with exercise. Observational studies in advanced cancer have demonstrated that physical function and patient-reported outcomes often deteriorate rapidly during cancer treatment [61,62,63]. Our findings suggest that exercise may help to attenuate the impact of treatment on symptom levels, while enhancing physical fitness, even in those receiving active chemotherapy. Furthermore, higher levels of physical functioning (e.g., performance status and mobility) have been associated with improved treatment tolerance in some cancer populations [64,65]. These results support consideration of exercise for individuals undergoing treatment for metastatic disease, with potential benefits for symptom control, physical fitness, and treatment outcomes.

Our analysis provides additional findings related to symptom status in the participants with metastatic cancer. The ESAS scores of the ACE participants were generally low compared to other datasets [60,66], suggesting those opting to participate in the ACE program may have had a lower symptom burden than typically seen. Moreover, while benefits were found for other patient-reported outcomes (i.e., FACT, EQ VAS), no significant changes were found in ESAS scores following the 12-week exercise program. One explanation is that the ESAS may lack sensitivity to improvement in patients with stable disease presenting with low symptom burden. In ACE, we also note the small proportion of participants who dropped out of the study had worse symptom scores at baseline than those who completed the study. Cancer-related symptoms, particularly fatigue, have been commonly identified as key barriers to exercise participation in individuals with advanced cancer [16,18,19,20,21]. These findings further reinforce the need for symptom assessment and tailored exercise support, as well as interdisciplinary support for comprehensive symptom management, particularly for those with high symptom burden [67].

The high study completion and exercise attendance rates in this analysis support feasibility and effectiveness of community-based exercise programming for the subgroup of ACE participants with metastatic cancer. It is important to note the low rate of adverse events among these participants in the ACE study. Previous reviews have demonstrated similarly low rates of adverse events in individuals with advanced disease participating in structured exercise trials [11,13,68]. However, our understanding of adverse events related to exercise is limited due to absent or insufficient reporting of exercise-related harms in the exercise oncology literature, as well as limited representation of diverse and advanced cancer types [13,69,70]. The risk of adverse events in participants with metastatic cancer is anticipated given the high potential for disease progression and unpredictable symptom burden in this population. However, the overall risk remains quite low, particularly in comparison to the potential for benefit from exercise in these individuals. Consideration of these known risks is crucial when designing and implementing exercise programming in oncology. Key practices to address the risk of adverse events include comprehensive screening and routine monitoring, tailored exercise support (e.g., medical supervision) and modifications, and training of exercise professionals in adverse event identification, management and reporting [71,72,73].

Finally, our exploratory analyses yield interesting results regarding subgroup findings according to exercise training type. Improvements on the FACT measure were strongest for participants who were engaging in the in-person group personal training, followed closely by those in the virtual circuit training. These findings suggest that exercise-related factors may influence the degree and type of benefits obtained by participants. The delivery of the ACE program took place in real-world settings and conditions, including virtual formats following the initial lockdown due to COVID-19. Though the ACE program was delivered at most sites as a full-body circuit training program, participants were shown modifications for each exercise to suit individual needs and capabilities. Selected sites also offered group-based personal training options for participants requiring more tailored exercise support. Thus, flexibility in exercise prescription and delivery may be an important consideration to support engagement and optimize the benefits to participants.

## 5. Limitations

Limitations in the study population include the high percentage of breast cancer compared to other cancer types, as well as the relatively low symptom burden of the participants. However, the study had considerable representation of individuals with non-breast cancer types and with higher symptom burden, allowing generalizability of these findings to a heterogenous population of metastatic cancer. ACE participants were mainly of European ethnic origin, and there were low proportions of individuals of visible minorities and Indigenous populations [74]. Personal factors, such as cultural and ethnic origin, race, and gender identity, require important consideration as they may influence disparities in physical activity engagement and access to exercise-related information and support [75,76,77]. Future exercise oncology research should consider more deliberate identification and representation of diverse participant profiles, and investigation of factors influencing key outcomes. Another study limitation is the use of the ESAS measure to assess cancer-related symptoms. Though this measure has strong clinical use [34], it may be limited as a tool to capture changes in symptom burden over time. The use of other self-reported and objective measures strengthened the findings of the ACE study. Finally, there were high rates of missing data for the physical fitness measures during the COVID-19 lockdown. However, a key learning from the ACE study is how to adapt and implement study assessments and exercise programming for individuals with metastatic cancer under real-world conditions.

## 6. Conclusions

The ACE study highlights the feasibility and benefit of cancer-specific community-based exercise programming for individuals diagnosed with different types of metastatic cancer. Due to the large sample size of the ACE study, we have the opportunity to further explore factors associated with adherence and response to exercise across different cancer types and stages to help inform clinical programming recommendations.

## Figures and Tables

**Table 1:** Baseline sociodemographic characteristics of participants with metastatic cancer enrolled in the Alberta Cancer Exercise program (N = 306).

Sociodemographic Characteristic	ACE-Met Participants		ACE-Met Participants Completing Study		ACE-Met Participants Not Completing Study	
	N = 306		N = 274		N = 32	
	*Mean/n*	*SD/%*	*Mean/n*	*SD/%*	*Mean/n*	*SD/%*
Age at diagnosis, mean, SD	57.6	11.9	58.0	12.0	54.1	11.0
Age category at diagnosis						
Young adult (18–39)	23	7.5	20	7.3	3	9.4
Middle-aged (40–64)	191	62.4	169	61.7	22	68.8
Older adult (65+)	92	30.1	85	31.0	7	21.9
Biological sex						
Female	200	65.4	181	66.1	19	59.4
Male	106	34.6	93	33.9	13	40.6
Ethnicity						
North American—Indigenous	2	0.7	2	0.7	0	0.0
North American—Other	4	1.3	4	1.5	0	0.0
European	232	75.8	209	76.3	23	71.9
Latin, Central, or South American or Caribbean	4	1.3	4	1.5	0	0.0
African	2	0.7	2	0.7	0	0.0
Asian or Oceanian	28	9.2	23	8.4	5	15.6
Multiple or other	18	5.9	18	6.6	0	0.0
Missing or unknown	16	5.2	12	4.4	4	12.5
Marital status						
Married or common-law	226	73.9	201	73.4	25	78.1
Divorced, separated, widowed	53	17.3	48	17.5	5	15.6
Single (never married)	27	8.8	25	9.1	2	6.3
Highest level of education						
Did not complete college/university	102	33.3	86	31.4	16	50.0
Completed college/university or higher	204	66.7	188	68.6	16	50.0
Income						
≤$60,000	95	31.0	89	32.5	6	18.8
$60,000 to $99,999	82	26.8	70	25.5	12	37.5
>$100,000	98	32.0	89	32.5	9	28.1
Missing	31	10.1	26	9.5	5	15.6
Employment						
Disability or temporarily unemployed	139	45.4	124	45.3	15	46.9
Retired	100	32.7	92	33.6	8	25.0
Homemaker	12	3.9	10	3.6	2	6.3
Part-time or full-time	55	18.0	48	17.5	7	21.9
Smoking						
Non-smoker	174	56.9	152	55.5	22	68.8
Ex-smoker	118	38.6	109	39.8	9	28.1
Occasional or regular smoker	14	4.6	13	4.7	1	3.1
Drinking						
Non- or ex-drinker	77	25.2	71	25.9	6	18.8
Occasional or social drinker	219	71.6	193	70.4	26	81.3
Daily drinker	10	3.3	10	3.6	0	0.0
BMI, mean, SD	28.0	6.2	28.0	6.0	27.9	7.2
BMI category						
Normal/underweight	98	32.0	86	31.4	12	37.5
Overweight	109	35.6	101	36.9	8	25.0
Obese	99	32.4	87	31.8	12	37.5
Number of comorbidities						
None	86	28.1	75	27.4	11	34.4
One	113	36.9	102	37.2	11	34.4
Two or more	107	35.0	97	35.4	10	31.3
Type of comorbidity						
Arthritis	146	47.7	133	48.5	13	40.6
Cardiovascular	75	24.5	65	23.7	10	31.3
Mental health	42	13.7	38	13.9	4	12.5
Metabolic	32	10.5	28	10.2	4	12.5
Other	76	24.8	73	26.6	3	9.4

ACE-Met: Alberta Cancer Exercise participants with metastatic cancer; BMI: body mass index; SD: standard deviation.

**Table 2:** Baseline cancer-related characteristics of participants with metastatic cancer enrolled in the Alberta Cancer Exercise program (N = 306).

Cancer-Related Characteristic	ACE-Met Participants		ACE-Met Participants Completing Study		ACE-Met Participants Not Completing Study	
	N = 306		N = 274		N = 32	
	*Mean/n*	*SD/%*	*Mean/n*	*SD/%*	*Mean/n*	*SD/%*
Primary cancer type						
Breast	103	33.7	89	32.5	14	43.8
Lung	33	10.8	31	11.3	2	6.3
Digestive	46	15.0	36	13.1	10	31.3
Blood	19	6.2	19	6.9	0	0.0
Gynecologic	30	9.8	28	10.2	2	6.3
Genitourinary	51	16.7	48	17.5	3	9.4
Head and neck	10	3.3	10	3.6	0	0.0
Skin/melanoma	7	2.3	7	2.6	0	0.0
Other	7	2.3	6	2.2	1	3.1
Number of organ sites affected by metastases, mean, SD	1.4	0.7	1.4	0.7	1.6	0.8
Number of organ sites affected by metastases category						
One	204	66.7	185	67.5	19	59.4
Two or more	102	33.3	89	32.5	13	40.6
Location of metastases						
Bone	137	44.8	122	44.5	15	46.9
Brain	31	10.1	25	9.1	6	18.8
Liver	88	28.8	77	28.1	11	34.4
Lung	79	25.8	66	24.1	13	40.6
Other distant site	62	20.3	58	21.2	4	12.5
Regional spread	12	3.9	12	4.4	0	0.0
Treatment status						
On	229	74.8	204	74.5	25	78.1
Off	77	25.2	70	25.5	7	21.9
Current treatment(s)						
Chemotherapy	110	35.9	92	33.6	18	56.3
Radiation	8	2.6	6	2.2	2	6.3
Hormone therapy	87	28.4	83	30.3	4	12.5
Biological/targeted/immune therapy	69	22.5	65	23.7	4	12.5
Other	4	1.3	4	1.5	0	0.0
Completed treatment(s)						
Surgery	165	53.9	147	53.6	18	56.3
Chemotherapy	181	59.2	159	58.0	22	68.8
Radiation	145	47.4	128	46.7	17	53.1
Hormone therapy	58	19.0	50	18.2	8	25.0
Biological/targeted/immune therapy	27	8.8	25	9.1	2	6.3
Stem cell transplant	9	2.9	9	3.3	0	0.0
Other	7	2.3	7	2.6	0	0.0
No prior therapy	7	2.3	5	1.8	2	6.3

ACE-Met: Alberta Cancer Exercise participants with metastatic cancer; SD: standard deviation.

**Table 3:** Exercise-related characteristics of participants with metastatic cancer enrolled in the Alberta Cancer Exercise program (N = 306).

Exercise-Related Characteristic	ACE-Met Participants		ACE-Met Participants Completing Study		ACE-Met Participants Not Completing Study	
	N = 306		N = 274		N = 32	
	*Mean/n*	*SD/%*	*Mean/n*	*SD/%*	*Mean/n*	*SD/%*
PA minutes per week at baseline, mean, SD	90.2	157.9	93.6	160.3	60.9	134.8
PA classification at baseline						
Sedentary (0 min)	151	49.3	137	50.0	14	43.8
Insufficiently active (<150 min)	88	28.8	73	26.6	15	46.9
Active (150 min or higher)	66	21.6	63	23.0	3	9.4
Missing	1	0.3	1	0.4	0	0.0
PA stages of change at baseline						
Precontemplation	5	1.6	4	1.5	1	3.1
Contemplation	71	23.2	61	22.3	10	31.3
Preparation	96	31.4	85	31.0	11	34.4
Decision/Action	11	3.6	11	4.0	0	0.0
Maintenance	37	12.1	36	13.1	1	3.1
Missing	86	28.1	77	28.1	9	28.1
Exercise location						
Calgary	140	45.8	129	47.1	11	34.4
Edmonton	130	42.5	114	41.6	16	50.0
North Zone (Fort McMurray, Grand Prairie, Red Deer)	21	6.9	18	6.6	3	9.4
South Zone (Lethbridge, Medicine Hat)	15	4.9	13	4.7	2	6.3
Type of exercise class						
In-person circuit training	133	43.5	116	42.3	17	53.1
In-person personal training	100	32.7	89	32.5	11	34.4
Virtual circuit training	73	23.9	69	25.2	4	12.5

ACE-Met: Alberta Cancer Exercise participants with metastatic cancer; PA: physical activity; SD: standard deviation.

**Table 4:** Baseline scores and associated changes at 12 weeks for Alberta Cancer Exercise participants with metastatic cancer who completed 12-week study (N = 274).

Outcome Measures	ACE-Met Participants Completing Study				
	n	Baseline Score *Mean (sd)*	12-Week Score *Mean (sd)*	12-Week Change *Mean (95% CI)*	*p*-Value
FACT					
FACT-G total scale (0–108)	268	74.5 (14.2)	75.5 (15.3)	1.0 (−0.3, 2.2)	0.010 *
FACT Trial Outcome Index (0–108)	269	72.4 (17.9)	75.7 (19.0)	3.4 (1.6, 5.1) *	<0.001
FACT Fatigue subscale (0–52)	269	34.8 (10.4)	37.1 (10.5)	2.4 (1.4, 3.3) *	<0.001
FACT-F total scale (0–160)	268	109.3 (23.0)	112.7 (24.3)	3.4 (1.3, 5.4) *	<0.001
ESAS					
Physical (0–60)	268	10.0 (8.9)	10.1 (8.9)	0.1 (−0.9, 1.1)	0.870
Emotional (0–20)	268	3.5 (4.2)	3.5 (4.2)	0.0 (−0.4, 0.4)	0.873
Wellbeing (0–10)	268	3.4 (2.5)	3.3 (2.5)	−0.1 (−0.4, 0.3)	0.659
Total symptom distress (0–90)	268	16.9 (13.1)	17.0 (13.0)	0.1 (−1.3, 1.4)	0.873
EQ VAS (0–100)	270	64.1 (18.2)	69.4 (18.1)	5.3 (3.2, 7.3) *†	<0.001
Physical activity minutes per week	269	94.2 (161.3)	161.9 (183.8)	67.7 (48.7, 86.8) *†	<0.001
Physical fitness					
30 s sit-to-stand	227	13.5 (5.2)	16.5 (5.8)	2.9 (2.4, 3.4) *†	<0.001
One-legged stance	222	25.2 (15.9)	29.6 (15.7)	4.4 (2.9, 5.9) *	<0.001
6MWT	173	525.4 (113.3)	558.7 (113.6)	33.2 (22.2, 44.3) *†	<0.001
2 min step test	50	74.0 (24.4)	84.1 (25.3)	10.1 (5.7, 14.6) *†	<0.001
Right shoulder AROM	235	148.3 (11.8)	150.4 (11.7)	2.1 (1.0, 3.3) *	<0.001
Left shoulder AROM	235	146.3 (12.9)	148.7 (12.2)	2.4 (1.2, 3.7) *	<0.001

ACE-Met: Alberta Cancer Exercise participants with metastatic cancer; AROM: active range of motion; CI: confidence interval; ESAS: Edmonton Symptom Assessment System; FACT-F: Functional Assessment of Cancer Therapy-Fatigue; FACT-G: Functional Assessment of Cancer Therapy-General; SD: standard deviation; VAS: visual analog scale. Higher scores on FACT scales and subscales indicate better quality of life; higher scores on ESAS items indicate higher symptom burden; higher scores on EQ VAS indicate better health. * Significant with Wilcoxon signed rank tests (*p* < 0.01). † Meeting minimal important difference for improvement.

## Data Availability

The original contributions presented in this study are included in the article/Supplementary Material. Further inquiries can be directed to the corresponding author.

## Supplementary Materials

The following supporting information can be downloaded at https://www.mdpi.com/article/10.3390/curroncol32100560/s1. File S1: Baseline symptom-related and physical fitness measures of participants with metastatic cancer enrolled in the Alberta Cancer Exercise study (N = 306); File S2: Baseline ESAS item scores for participants with metastatic cancer enrolled in the Alberta Cancer Exercise study (N = 306) *; File S3: Proportion of responses by level of severity on EQ-5D-5L at baseline for participants with metastatic cancer enrolled in the Alberta Cancer Exercise study (N = 306); File S4: Proportion of responses by level of severity on EQ-5D-5L dimensions at baseline and at 12 weeks for Alberta Cancer Exercise participants with metastatic cancer who completed 12-week study (n = 274) *; File S5: Baseline scores and associated changes at 12 weeks for Alberta Cancer Exercise participants with metastatic cancer who completed 12-week study, by biological sex (female: n = 181; male: n = 93); File S6: Baseline scores and associated changes at 12 weeks for Alberta Cancer Exercise participants with metastatic cancer who completed 12-week study, by chemotherapy treatment status (on current chemotherapy: n = 92; off chemotherapy: n = 182); File S7: Baseline symptom and physical fitness measures and associated changes at 12 weeks for Alberta Cancer Exercise participants with metastatic cancer who completed 12-week study, by exercise training type (in-person circuit training: n = 116, in-person personal training: n = 89; virtual circuit training: n = 69).
