# Supplementary material for: Baseline Characteristics of Individuals with Metastatic Cancer Enrolled in the Alberta Cancer Exercise Study and 12-Week Findings for Symptom-Related and Physical Fitness Measures
Source: Curr Oncol. 2025 Oct 7;32(10):560. doi: 10.3390/curroncol32100560 (PMC12562936; doi:10.3390/curroncol32100560)
Supplement: Supplementary file 1 [file curroncol-32-00560-s001.zip › curroncol-3843782-supplementary.pdf]

## Supplementary Files

**Manuscript:** Baseline characteristics of individuals with metastatic cancer enrolled in the Alberta Cancer Exercise Study and 12-week findings for symptom-related and physical fitness measures

**Supplementary File S1.** Baseline symptom-related and physical fitness measures of participants with metastatic cancer enrolled in the Alberta Cancer Exercise study (N=306)

| **Symptom-Related and Physical Fitness Measures** | **ACE-Met Participants** N=306 | | | **ACE-Met Participants Completing Study** N=274 | | **ACE-Met Participants Not Completing Study** N=32 | |
|---|---|---|---|---|---|---|---|
| | *Mean / n* | *SD / %* | *n* | *Mean / n* | *SD / %* | *Mean / n* | *SD / %* |
| FACT scores, mean, SD | | | | | | | |
| Physical well-being subscale (0-28) | 20.8 | 4.8 | 305 | 21.0 | 4.8 | 18.7 | 4.3 |
| Social/family well-being subscale (0-28) | 19.1 | 4.4 | 304 | 19.3 | 4.3 | 17.2 | 4.6 |
| Emotional well-being subscale (0-24) | 17.3 | 4.5 | 305 | 17.5 | 4.4 | 15.3 | 4.6 |
| Functional well-being subscale (0-28) | 16.2 | 5.5 | 305 | 16.5 | 5.5 | 14.2 | 5.0 |
| Fatigue subscale (0-52) | 34.1 | 10.6 | 305 | 34.6 | 10.5 | 30.1 | 11.0 |
| Trial outcome index (0-108) | 71.1 | 18.3 | 305 | 72.1 | 18.1 | 63.0 | 18.4 |
| FACT-G total (0-108) | 73.4 | 14.6 | 304 | 74.3 | 14.3 | 65.3 | 14.8 |
| FACT-F total (0-160) | 107.6 | 23.7 | 304 | 109.0 | 23.3 | 95.4 | 24.1 |
| ESAS scores, mean, SD | | | | | | | |
| Physical (0-60) | 10.7 | 9.3 | 305 | 10.2 | 9.1 | 15.1 | 10.2 |
| Emotional (0-20) | 3.7 | 4.4 | 305 | 3.6 | 4.4 | 4.7 | 4.6 |
| Wellbeing (0-10) | 3.5 | 2.5 | 305 | 3.4 | 2.5 | 4.0 | 2.2 |
| Total symptom distress (0-90) | 17.9 | 13.8 | 305 | 17.2 | 13.5 | 23.7 | 14.6 |
| EQ VAS (0-100), mean, SD | 62.9 | 18.6 | 306 | 64.2 | 18.2 | 51.8 | 18.6 |
| One-legged stance (seconds), mean, SD | 25.0 | 16.0 | 292 | 25.0 | 16.0 | 25.0 | 16.3 |
| One-legged stance category | | | | | | | |
| Below norms | 147 | 48.0 | | 131 | 47.8 | 16 | 50.0 |
| Meeting / exceeding norms | 147 | 48.0 | | 132 | 48.2 | 15 | 46.9 |
| Missing | 12 | 3.9 | | 11 | 4.0 | 1 | 3.1 |
| 30-second sit-to-stand, mean, SD | 13.5 | 5.5 | 297 | 13.6 | 5.5 | 12.6 | 4.7 |
| 30-second sit-to-stand category | | | | | | | |
| Below norms* | 243 | 79.4 | | 215 | 78.5 | 28 | 87.5 |
| Meeting / exceeding norms* | 54 | 17.6 | | 52 | 19.0 | 2 | 6.3 |
| Missing | 9 | 2.9 | | 7 | 2.6 | 2 | 6.3 |

| | | | | | | | |
|---|---|---|---|---|---|---|---|
| 6MWT distance (metres), mean, SD | 528.0 | 112.3 | 243 | 527.5 | 113.9 | 532.1 | 101.0 |
| 2-minute step test, mean, SD | 72.6 | 23.0 | 60 | 73.2 | 23.5 | 64.0 | 10.2 |
| Grip strength (kg), mean, SD | 63.2 | 19.1 | 244 | 63.2 | 19.0 | 62.7 | 20.1 |
| Grip strength category | | | | | | | |
| Below norms* | 99 | 32.4 | | 86 | 31.4 | 13 | 40.6 |
| Meeting / exceeding norms* | 145 | 47.4 | | 131 | 47.8 | 14 | 43.8 |
| Missing | 62 | 20.3 | | 57 | 20.8 | 5 | 15.6 |
| Shoulder AROM (degrees), mean, SD | | | | | | | |
| Right | 148.0 | 12.1 | 303 | 147.9 | 11.9 | 149.4 | 13.1 |
| Left | 146.7 | 12.4 | 303 | 146.3 | 12.6 | 149.7 | 10.6 |

ACE-Met: Alberta Cancer Exercise participants with metastatic cancer; AROM: active range of motion; ESAS: Edmonton Symptom Assessment System; FACT-F: Functional Assessment of Cancer Therapy-Fatigue; FACT-G: Functional Assessment of Cancer Therapy-General; SD: standard deviation; VAS: visual analog scale.

*Age- and sex-matched norms.

Higher scores on FACT scales and subscales indicate better quality of life; higher scores on ESAS items indicate higher symptom burden; higher scores on EQ VAS indicate better health.

**Supplementary File S2.** Baseline ESAS item scores for participants with metastatic cancer enrolled in the Alberta Cancer Exercise study (N=306)*

| | Symptom Score | Frequency of Symptom Severity | | |
|---|---|---|---|---|
| **ESAS Item** | | No / mild (score 0-3) | Moderate (score 4-6) | Severe (score 7-10) |
| | *Mean (sd)* | *N (%)* | *N (%)* | *N (%)* |
| Pain | 2.0 (2.0) | 249 (81.6%) | 43 (14.1%) | 13 (4.2%) |
| Tiredness | 3.5 (2.6) | 171 (56.1%) | 82 (26.9%) | 52 (17.0%) |
| Drowsiness | 2.3 (2.5) | 224 (73.4%) | 54 (17.7%) | 29 (9.5%) |
| Nausea | 0.6 (1.4) | 290 (95.1%) | 10 (3.3%) | 5 (1.6%) |
| Appetite | 1.1 (2.0) | 270 (88.5%) | 24 (7.9%) | 11 (3.6%) |
| Shortness of breath | 1.2 (2.0) | 268 (87.9%) | 25 (8.2%) | 12 (3.9%) |
| Depression | 1.8 (2.3) | 249 (81.6%) | 36 (11.8%) | 20 (6.6%) |
| Anxiety | 1.9 (2.3) | 246 (80.7%) | 37 (12.1%) | 22 (7.2%) |
| Wellbeing | 3.5 (2.5) | 164 (53.8%) | 102 (33.4%) | 39 (12.8%) |

ESAS: Edmonton Symptom Assessment System.

Higher scores on ESAS items indicate higher symptom burden.

*Missing baseline ESAS data for n=1 (total N=305).

**Supplementary File S3.** Proportion of responses by level of severity on EQ-5D-5L at baseline for participants with metastatic cancer enrolled in the Alberta Cancer Exercise study (N=306)

| | **Frequency of Problem Severity** | | | | |
|---|---|---|---|---|---|
| **EQ-5D-5L Dimension (Level: 1-5)** | Level 1: No problems | Level 2: Slight problems | Level 3: Moderate problems | Level 4: Severe problems | Level 5: Extreme problems |
| | *N (%)* | *N (%)* | *N (%)* | *N (%)* | *N (%)* |
| Mobility | 175 (57.2%) | 83 (27.1%) | 41 (13.4%) | 7 (2.3%) | 0 (0.0%) |
| Self-care | 262 (85.6%) | 41 (13.4%) | 3 (1.0%) | 0 (0.0%) | 0 (0.0%) |
| Activity | 90 (29.4%) | 125 (40.8%) | 75 (24.5%) | 12 (3.9%) | 4 (1.3%) |
| Pain / discomfort | 70 (22.9%) | 164 (53.6%) | 64 (20.9%) | 7 (2.3%) | 1 (0.3%) |
| Anxiety / depression | 103 (33.7%) | 137 (44.8%) | 51 (16.7%) | 11 (3.6%) | 4 (1.3%) |
| **EQ VAS (Score: 0-100)** | VAS: 81-100 (100: Best health) | VAS: 61-80 | VAS: 41-60 | VAS: 21-40 | VAS: 0-20 (0: Worst health) |
| | *N (%)* | *N (%)* | *N (%)* | *N (%)* | *N (%)* |
| | 50 (16.3%) | 128 (41.8%) | 81 (26.5%) | 44 (14.4%) | 3 (1.0%) |

EQ-5D-5L: 5-level EQ-5D version; VAS: visual analog scale.

Higher levels on EQ-5D-5L dimensions indicate more severe problems; higher scores on EQ VAS indicate better health; 100: best health you can imagine; 0: worst health you can imagine.

**Supplementary File S4.** Proportion of responses by level of severity on EQ-5D-5L dimensions at baseline and at 12 weeks for Alberta Cancer Exercise participants with metastatic cancer who completed 12-week study (n=274)*

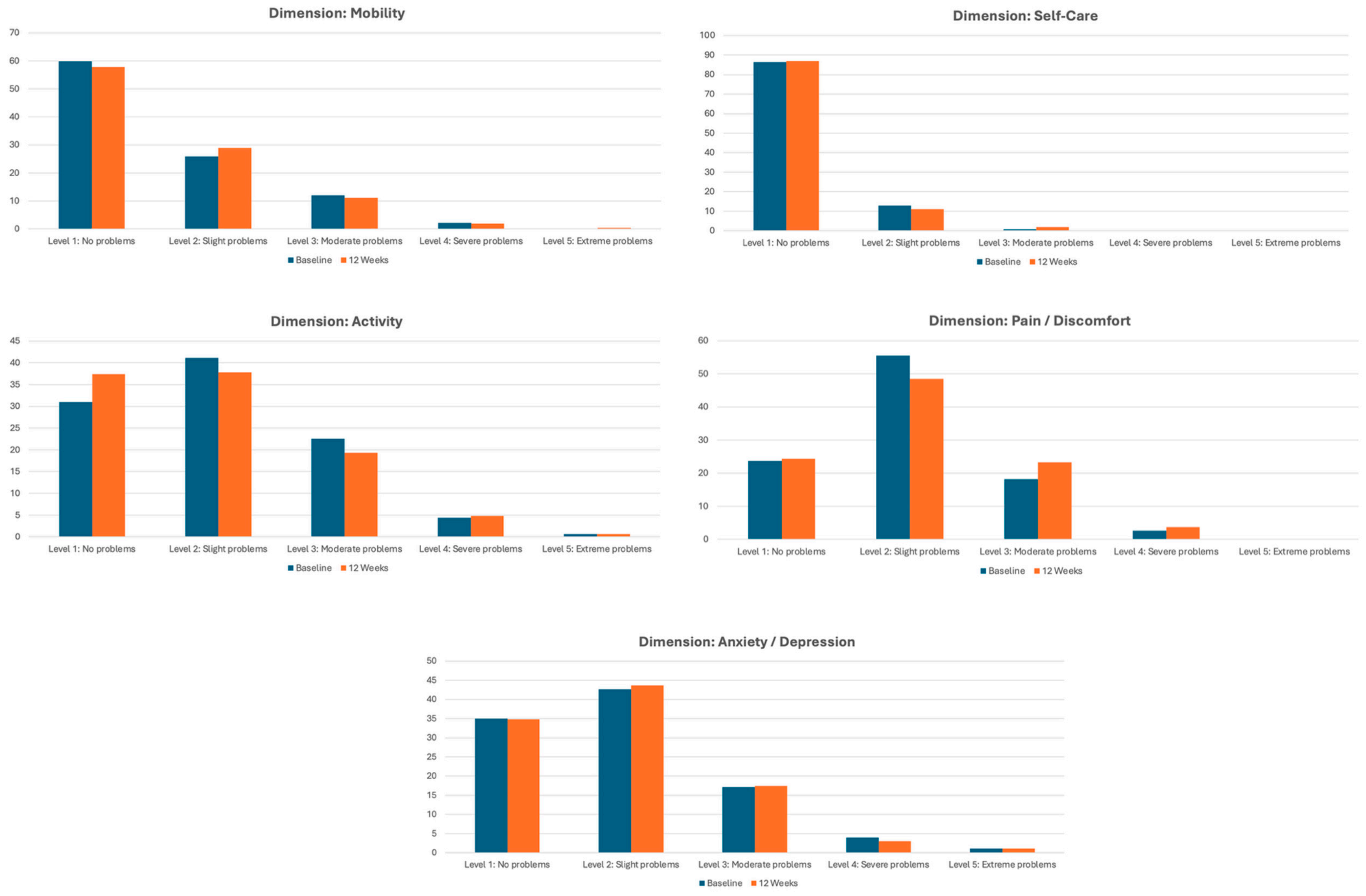


*Missing data for 4 participants at 12 weeks

**Supplementary File S5.** Baseline scores and associated changes at 12 weeks for Alberta Cancer Exercise participants with metastatic cancer who completed 12-week study, by biological sex (female: n=181; male: n=93)

| Outcome Measures | *Category 1:* **Female ACE-Met Participants** | | | | *Category 2:* **Male ACE-Met Participants** | | | |
|---|---|---|---|---|---|---|---|---|
| | Baseline score *Mean (sd)* | 12-week change *Mean (95% CI)* | p-value | *n* | Baseline score *Mean (sd)* | 12-week change *Mean (95% CI)* | p-value | *n* |
| FACT | | | | | | | | |
| FACT-G total scale (0-108) | 74.3 (14.1) | 0.5 (-1.2, 2.2) | 0.133 | 178 | 74.9 (14.4) | 2.0 (0.2, 3.8)* | 0.018 | 90 |
| FACT TOI (0-108) | 72.5 (17.7) | 2.6 (0.2, 4.9)* | 0.002 | 179 | 72.1 (18.3) | 5.0 (2.8, 7.1)*† | <0.001 | 90 |
| FACT Fatigue subscale (0-52) | 34.9 (10.0) | 1.9 (0.6, 3.3)* | <0.001 | 179 | 34.4 (11.3) | 3.2 (1.8, 4.6)*† | <0.001 | 90 |
| FACT-F total scale (0-160) | 109.3 (22.5) | 2.4 (-0.3, 5.2)* | 0.006 | 178 | 109.4 (24.0) | 5.2 (2.5, 7.9)* | <0.001 | 90 |
| ESAS | | | | | | | | |
| Physical (0-60) | 9.8 (9.0) | 0.5 (-0.8, 1.9) | 0.482 | 178 | 10.4 (8.6) | -0.8 (-2.0, 0.4) | 0.164 | 90 |
| Emotional (0-20) | 3.6 (4.4) | 0.2 (-0.3, 0.8) | 0.457 | 178 | 3.2 (4.0) | -0.3 (-0.8, 0.2) | 0.111 | 90 |
| Wellbeing (0-10) | 3.3 (2.5) | 0.1 (-0.3, 0.6) | 0.591 | 178 | 3.4 (2.4) | -0.5 (-0.9, -0.0) | 0.062 | 90 |
| Total symptom distress (0-90) | 16.8 (13.2) | 0.9 (-0.9, 2.7) | 0.333 | 178 | 17.1 (13.0) | -1.6 (-3.2, 0.1) | 0.060 | 90 |
| EQ VAS (0-100) | 65.8 (17.0) | 3.5 (1.0, 6.0)* | <0.001 | 179 | 60.7 (20.1) | 8.9 (5.3, 12.4)*† | <0.001 | 91 |
| PA minutes per week | 82.2 (143.5) | 67.6 (46.3, 88.9)*† | <0.001 | 178 | 117.5 (190.0) | 68.0 (29.7, 106.3)*† | <0.001 | 91 |
| Physical fitness | | | | | | | | |
| 30-second sit-to-stand | 13.1 (4.7) | 3.0 (2.4, 3.6)*† | <0.001 | 150 | 14.4 (6.2) | 2.7 (1.9, 3.6)*† | <0.001 | 77 |
| One-legged stance | 26.5 (15.6) | 4.3 (2.4, 6.2)* | <0.001 | 146 | 22.7 (16.3) | 4.7 (2.2, 7.1)* | <0.001 | 76 |
| 6MWT | 524.2 (107.0) | 33.9 (20.2, 47.6)*† | <0.001 | 110 | 527.7 (124.5) | 32.0 (13.0, 51.1)*† | <0.001 | 63 |
| 2-minute step test | 75.0 (25.7) | 8.9 (3.5, 14.2)*† | 0.002 | 37 | 71.2 (21.4) | 13.8 (4.7, 23.0)*† | 0.007 | 13 |
| Right shoulder AROM | 150.2 (13.0) | 1.7 (0.3, 3.2)* | 0.036 | 155 | 144.6 (11.8) | 3.0 (1.2, 4.8)* | 0.002 | 80 |
| Left shoulder AROM | 147.6 (13.0) | 2.1 (0.4, 3.8)* | 0.009 | 155 | 143.9 (12.2) | 3.0 (1.2, 4.8)* | <0.001 | 80 |

ACE-Met: Alberta Cancer Exercise participants with metastatic cancer; AROM: active range of motion; CI: confidence interval; ESAS: Edmonton Symptom Assessment System; FACT-F: Functional Assessment of Cancer Therapy-Fatigue; FACT-G: Functional Assessment of Cancer Therapy-General; PA: physical activity; SD: standard deviation; TOI: Trial Outcome Index; VAS: visual analog scale.

Higher scores on FACT scales and subscales indicate better quality of life; higher scores on ESAS items indicate higher symptom burden; higher scores on EQ VAS indicate better health.

*Significant with Wilcoxon signed-rank tests (p<0.05). †Meeting minimal clinically important difference for improvement.

**Supplementary File S6.** Baseline scores and associated changes at 12 weeks for Alberta Cancer Exercise participants with metastatic cancer who completed 12-week study, by chemotherapy treatment status (on current chemotherapy: n=92; off chemotherapy: n=182)

| **Outcome Measures** | ***Category 1:* ACE-Met Participants on Current Chemotherapy** | | | | ***Category 2:* ACE-Met Participants Off Current Chemotherapy** | | | |
|---|---|---|---|---|---|---|---|---|
| | Baseline score *Mean (sd)* | 12-week change *Mean (95% CI)* | p-value | *n* | Baseline score *Mean (sd)* | 12-week change *Mean (95% CI)* | p-value | *n* |
| FACT | | | | | | | | |
| FACT-G total scale (0-108) | 76.9 (12.6) | -0.9 (-3.3, 1.4) | 0.867 | 89 | 73.3 (14.8) | 1.9 (0.4, 3.4)* | 0.001 | 179 |
| FACT TOI (0-108) | 73.8 (16.9) | 0.9 (-2.7, 4.6) | 0.283 | 89 | 71.7 (18.3) | 4.6 (2.7, 6.4)* | <0.001 | 180 |
| FACT Fatigue subscale (0-52) | 35.4 (9.9) | 1.0 (-1.0, 3.1) | 0.131 | 89 | 34.5 (10.7) | 3.0 (2.0, 4.1)*† | <0.001 | 180 |
| FACT-F total scale (0-160) | 112.3 (20.7) | 0.1 (-4.0, 4.2) | 0.514 | 89 | 107.8 (23.9) | 5.0 (2.7, 7.2)* | <0.001 | 179 |
| ESAS | | | | | | | | |
| Physical (0-60) | 8.4 (8.1) | 1.9 (0.1, 3.8)* | 0.036 | 89 | 10.8 (9.1) | -0.8 (-1.9, 0.3) | 0.079 | 179 |
| Emotional (0-20) | 2.7 (3.8) | 0.4 (-0.3, 1.2) | 0.329 | 89 | 3.8 (4.3) | -0.1 (-0.6, 0.3) | 0.975 | 179 |
| Wellbeing (0-10) | 3.0 (2.6) | 0.5 (-0.2, 1.1) | 0.170 | 89 | 3.6 (2.4) | -0.3 (-0.8, 0.1) | 0.130 | 179 |
| Total symptom distress (0-90) | 14.0 (11.8) | 2.8 (0.1, 5.5)* | 0.031 | 89 | 18.3 (13.5) | -1.3 (-2.7, 0.1) | 0.061 | 179 |
| EQ VAS (0-100) | 65.3 (16.4) | 1.7 (-2.0, 5.3) | 0.076 | 90 | 63.5 (19.1) | 7.1 (4.6, 9.6)*† | <0.001 | 180 |
| PA minutes per week | 94.5 (177.6) | 54.4 (23.3, 85.4)*† | <0.001 | 90 | 94.0 (152.9) | 74.4 (50.4, 98.5)*† | <0.001 | 179 |
| Physical fitness | | | | | | | | |
| 30-second sit-to-stand | 13.5 (4.8) | 3.0 (2.0, 4.0)*† | <0.001 | 75 | 13.6 (5.5) | 2.9 (2.3, 3.4)*† | <0.001 | 152 |
| One-legged stance | 26.0 (15.6) | 3.1 (0.4, 5.9)* | 0.011 | 72 | 24.8 (16.0) | 5.0 (3.2, 6.8)* | <0.001 | 150 |
| 6MWT | 531.8 (122.3) | 32.0 (11.2, 52.9)*† | <0.001 | 57 | 522.3 (114.7) | 33.9 (20.8, 46.9)*† | <0.001 | 116 |
| 2-minute step test | 73.8 (26.4) | 5.3 (-3.1, 13.8) | 0.109 | 15 | 74.0 (24.0) | 12.2 (6.8, 17.6)*† | <0.001 | 35 |
| Right shoulder AROM | 150.0 (11.2) | 1.7 (-0.4, 3.9) | 0.161 | 75 | 147.5 (12.0) | 2.3 (1.0, 3.7)* | 0.001 | 160 |
| Left shoulder AROM | 147.6 (12.0) | 1.8 (-0.3, 3.9) | 0.093 | 75 | 145.7 (13.2) | 2.7 (1.1, 4.2)* | <0.001 | 160 |

ACE-Met: Alberta Cancer Exercise participants with metastatic cancer; AROM: active range of motion; CI: confidence interval; ESAS: Edmonton Symptom Assessment System; FACT-F: Functional Assessment of Cancer Therapy-Fatigue; FACT-G: Functional Assessment of Cancer Therapy-General; PA: physical activity; SD: standard deviation; TOI: Trial Outcome Index; VAS: visual analog scale. Higher scores on FACT scales and subscales indicate better quality of life; higher scores on ESAS items indicate higher symptom burden; higher scores on EQ VAS indicate better health. *Significant with Wilcoxon signed-rank tests (p<0.05). †Meeting minimal clinically important difference for improvement.

**Supplementary File S7.** Baseline symptom and physical fitness measures and associated changes at 12 weeks for Alberta Cancer Exercise participants with metastatic cancer who completed 12-week study, by exercise training type (in-person circuit training: n=116, in-person personal training: n=89; virtual circuit training: n=69)

| Outcome Measures | *Category 1:* ACE-Met Participants in Circuit Training (In-Person) | | | | *Category 2:* ACE-Met Participants in Personal Training (In-Person) | | | | *Category 3:* ACE-Met Participants in Circuit Training (Virtual) | | | |
|---|---|---|---|---|---|---|---|---|---|---|---|---|
| | Baseline score *Mean (sd)* | 12-week change *Mean (95% CI)* | p-value | *n* | Baseline score *Mean (sd)* | 12-week change *Mean (95% CI)* | p-value | *n* | Baseline score *Mean (sd)* | 12-week change *Mean (95% CI)* | p-value | *n* |
| FACT | | | | | | | | | | | | |
| FACT-G total scale (0-108) | 74.4 (13.3) | -0.1 (-2.2, 2.0) | 0.848 | 113 | 75.9 (14.2) | 1.9 (-0.1, 4.0)* | 0.006 | 87 | 73.0 (15.5) | 1.6 (-0.9, 4.1)* | 0.046 | 68 |
| FACT TOI (0-108) | 72.8 (17.1) | 1.9 (-0.8, 4.6) | 0.114 | 114 | 72.8 (18.2) | 4.8 (1.8, 7.7)* | <0.001 | 87 | 71.0 (18.9) | 4.0 (0.4, 7.6)* | <0.001 | 68 |
| FACT Fatigue subscale (0-52) | 35.2 (10.0) | 1.7 (0.2, 3.3)* | 0.022 | 114 | 34.6 (10.6) | 3.0 (1.3, 4.7)*† | <0.001 | 87 | 34.3 (11.0) | 2.6 (0.6, 4.6)* | <0.001 | 68 |
| FACT-F total scale (0-160) | 109.7 (21.6) | 1.6 (-1.6, 4.9) | 0.225 | 113 | 110.4 (23.5) | 4.9 (1.5, 8.4)* | <0.001 | 87 | 107.3 (24.7) | 4.2 (0.1, 8.2)* | 0.002 | 68 |
| ESAS | | | | | | | | | | | | |
| Physical (0-60) | 9.5 (8.4) | 1.4 (-0.1, 3.0)* | 0.039 | 114 | 10.8 (9.2) | -1.6 (-3.1, -0.1)* | 0.031 | 86 | 10.0 (9.2) | 0.0 (-2.1, 2.1) | 0.359 | 68 |
| Emotional (0-20) | 3.2 (3.9) | 0.3 (-0.3, 0.9) | 0.486 | 114 | 3.3 (3.9) | -0.1 (-0.7, 0.4) | 0.500 | 86 | 4.2 (5.2) | -0.1 (-1.1, 0.8) | 0.666 | 68 |
| Wellbeing (0-10) | 3.1 (2.4) | 0.0 (-0.5, 0.6) | 0.959 | 114 | 3.4 (2.4) | -0.2 (-0.8, 0.3) | 0.608 | 86 | 3.8 (2.5) | -0.1 (-1.0, 0.8) | 0.840 | 68 |
| Total symptom distress (0-90) | 15.9 (12.7) | 1.7 (-0.4, 3.9) | 0.067 | 114 | 17.4 (12.9) | -2.0 (-3.9, -0.0) | 0.066 | 86 | 18.0 (14.1) | -0.2 (-3.1, 2.7) | 0.396 | 68 |
| EQ VAS (0-100) | 64.5 (17.3) | 4.3 (1.1, 7.6)* | 0.003 | 115 | 65.2 (16.2) | 6.4 (3.1, 9.8)*† | <0.001 | 87 | 62.1 (22.1) | 5.4 (1.0, 9.8)*† | 0.007 | 68 |
| PA minutes per week | 104.9 (156.9) | 67.2 (35.9, 98.6)*† | <0.001 | 115 | 69.8 (127.2) | 71.1 (37.9, 104.3)*† | <0.001 | 86 | 106.9 (201.2) | 64.4 (29.4, 99.3)*† | <0.001 | 68 |
| Physical fitness | | | | | | | | | | | | |
| 30-second sit-to-stand | 15.3 (5.3) | 3.3 (2.3, 4.3)*† | <0.001 | 87 | 12.3 (5.4) | 3.2 (2.5, 3.9)*† | <0.001 | 79 | 12.5 (5.4) | 2.0 (1.1, 2.9)*† | <0.001 | 61 |
| One-legged stance | 24.8 (16.6) | 5.4 (2.7, 8.2)* | <0.001 | 85 | 23.4 (15.0) | 4.6 (2.3, 6.9)* | <0.001 | 76 | 27.8 (15.8) | 2.8 (0.1, 5.4) | 0.071 | 61 |

| | | | | | | | | | | | | |
|---|---|---|---|---|---|---|---|---|---|---|---|---|
| 6MWT | 557.2 (108.9) | 33.8 (16.0, 51.7)*† | <0.001 | 88 | 486.1 (107.5) | 37.1 (23.9, 50.3)*† | <0.001 | 76 | 546.4 (112.4) | -4.8 (-63.2, 53.6) | 0.767 | 9§ |
| 2-minute step test | n/a | n/a | | 0§ | n/a | n/a | | 0§ | 74.0 (24.5) | 10.2 (5.7, 14.6)*† | <0.001 | 50 |
| Right shoulder | 149.0 (12.4) | 1.4 (-0.6, 3.3) | 0.195 | 91 | 147.0 (12.7) | 1.9 (0.0, 3.8) | 0.066 | 82 | 149.0 (9.4) | 2.2 (0.6, 3.8)* | 0.002 | 62 |
| Left shoulder | 145.0 (14.1) | 3.0 (0.8, 5.2)* | 0.014 | 91 | 145.1 (13.1) | 1.9 (-0.4, 4.2)* | 0.034 | 82 | 149.9 (9.7) | 3.6 (1.5, 5.7)* | 0.0074 | 62 |

ACE-Met: Alberta Cancer Exercise participants with metastatic cancer; AROM: active range of motion; CI: confidence interval; ESAS: Edmonton Symptom Assessment System; FACT-F: Functional Assessment of Cancer Therapy-Fatigue; FACT-G: Functional Assessment of Cancer Therapy-General; PA: physical activity; SD: standard deviation; TOI: Trial Outcome Index; VAS: visual analog scale.

Higher scores on FACT scales and subscales indicate better quality of life; higher scores on ESAS items indicate higher symptom burden; higher scores on EQ VAS indicate better health.

*Significant with Wilcoxon signed-rank tests ($p<0.05$). †Meeting minimal clinically important difference for improvement.

§The 6MWT was measured only in virtual participants able to attend in-person testing; the 2-minute step test was not part of the test battery for in-person participants.
